# The interplay between antimicrobial resistance, heavy metal pollution, and the role of microplastics

**DOI:** 10.3389/fmicb.2025.1550587

**Published:** 2025-02-28

**Authors:** Igori Balta, Joanne Lemon, Anna Gadaj, Iuliana Cretescu, Ducu Stef, Ioan Pet, Lavinia Stef, David McCleery, Alastair Douglas, Nicolae Corcionivoschi

**Affiliations:** ^1^Faculty of Bioengineering of Animal Resources, University of Life Sciences King Mihai I from Timisoara, Timisoara, Romania; ^2^Chief Scientific Adviser’s Office, Department of Agriculture, Environment and Rural Affairs for Northern Ireland, Belfast, United Kingdom; ^3^Chemical Surveillance Branch, Veterinary Sciences Division, Agri-Food and Biosciences Institute, Belfast, United Kingdom; ^4^Department of Functional Sciences, Faculty of Medicine, Victor Babes University of Medicine and Pharmacy, Timisoara, Romania; ^5^Faculty of Food Engineering, University of Life Sciences King Mihai I from Timisoara, Timisoara, Romania; ^6^Bacteriology Branch, Veterinary Sciences Division, Agri-Food and Biosciences Institute, Belfast, United Kingdom; ^7^Veterinary Sciences Division, Agri-Food and Biosciences Institute, Belfast, United Kingdom; ^8^Academy of Romanian Scientists, Bucharest, Romania

**Keywords:** antimicrobial resistance, bacteria, heavy metals, environmental pollution, microplastics

## Abstract

Environmental pollution with heavy metals (HMs) and microplastics (MPs) could enhance the global health challenge antimicrobial resistance (AMR). Herein, we explore the complicated mechanics of how HMs, MPs, and AMR are interlinked within microbial ecosystems, as well as the co-selection and cross-resistance mechanisms. Unlike antibiotics, HMs have influenced microbial evolution for billions of years, promoting resistance mechanisms that predate antibiotic resistance genes (ARGs). At the same time, this conundrum is further complicated by the pervasive spread of MPs in the aquatic and terrestrial environments, acting as substrates for bacterial pathogenic biofilms and accelerates the horizontal gene transfer (HGT) of ARGs and heavy metal resistance genes (MRGs). This review highlights that HMs such as lead (Pb), mercury (Hg), arsenic (As), chromium (Cr), cadmium (Cd), and nickel (Ni) have persistently selected for resistance traits through efflux systems and genetic co-regulation. Together, these interactions are amplified by MPs that create genetic exchange hotspots due to biofilm formation. These dynamics are modulated by organic matter, which serves both as a nutrient source and a mediator of HM bioavailability, directly influencing ARG abundance. Soil and water ecosystems, including riverine systems and landfill leachate, are reservoirs for ARGs and ARG–MRG combinations, with notable contributions originating from anthropogenic activities. This review also emphasizes the urgent need for integrated environmental and public health strategies to mitigate pollutant-driven AMR. This work seeks to approach HMs and MPs as synergistic drivers of AMR such that both HMs and MPs are upstream (causes) levers, a foundation from which future research on sustainable environmental management practices and health policy (One Health Approach), aimed at curbing the spread of resistance determinants can proceed.

## 1 Introduction

According to the leading health and agriculture authorities, antimicrobial resistance (AMR) is rapidly emerging as one of the most serious global health crises, with diseases caused by antibiotic resistance bacteria (ARB) projected to surpass cancer as the leading cause of human mortality (Fu et al., 2023; Engin et al., 2023; Zieliński et al., 2021). Antibiotic resistance arises through two primary mechanisms, the intrinsic resistance and the acquired resistance (Nguyen et al., 2023). The intrinsic, or natural resistance, is present in bacterial genomes structural genes. Contrariwise, the acquired resistance is accumulated through horizontal gene transfer (HGT), transformation, transposition, and conjugation (Bunduruș et al., 2023). HGT is key in spreading antibiotic resistance among bacterial populations, including pathogenic ones, in both environmental and clinical settings (Nguyen et al., 2023; Bunduruș et al., 2023). This problem adds to environmental pollution, as it increases the generation of reactive oxygen species (ROS) that can directly orient stress-induced mutations in bacterial chromosomal DNA and thus fuel the constant evolution and subsequent outlasting of the resistance mechanisms (Nguyen et al., 2023).

**Figure 1:**
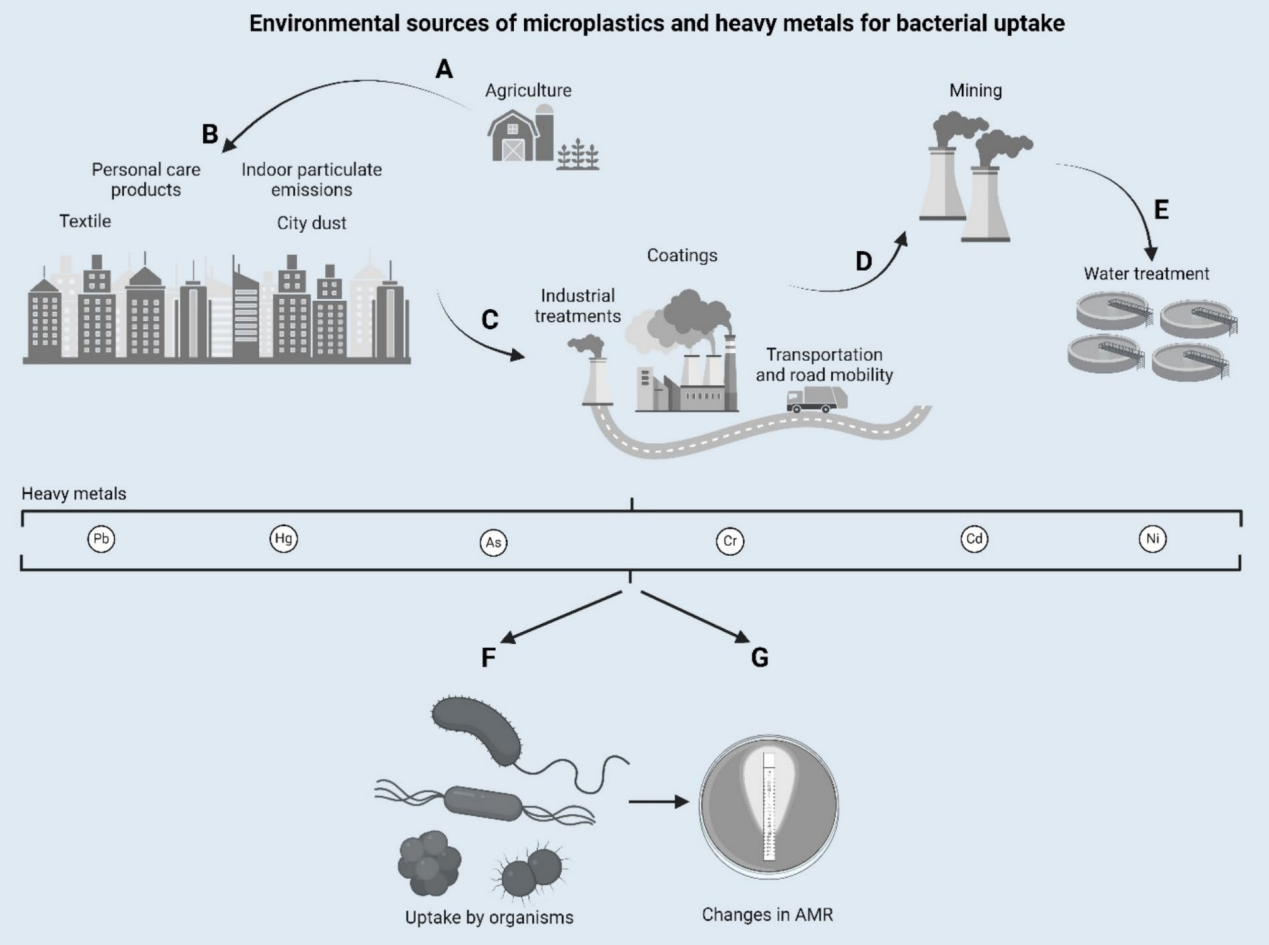
Environmental sources of microplastics and heavy metals for bacterial uptake. Designed with Biorender.com.

Although the role of antibiotics in propagating AMR has been extensively studied, emerging evidence highlights the contribution of environmental pollutants, such as microplastics (MPs) and heavy metals (HMs), to this phenomenon (Figure 1). It turns out that these pollutants, previously thought to be passive contaminants, were in fact playing an active role in driving the evolution of microbial resistance dynamics, amplifying the major threats posed by AMR toward public and environmental health (Engin et al., 2023).

Unlike antibiotics, metals have existed biologically since Earth’s formation and the Great Oxidation Event approximately 2.4 billion years ago (Vats et al., 2022). Interestingly, while antibiotic resistance (AR) was identified earlier, evidence suggests metal resistance predates it. For instance, mercury (Hg) resistance transposons, resembling contemporary Tn21-like transposons, but notably lacking antibiotic resistance genes (ARGs), have been isolated from bacteria predating the antibiotic era (Vats et al., 2022). Similarly, whole-genome sequencing of “pre-antibiotic era” bacteria, revived from long-term storage, has revealed the presence of multiple metal resistance genes (MRGs) (Vats et al., 2022; Dunne et al., 2017). These findings strongly indicate that metal resistance is an inherent feature of microbial communities exposed to heavy metals (HMs) throughout history. Moreover, this ancient metal resistance likely shaped the evolutionary trajectory of AMR, potentially accelerating its emergence and spread in the modern era (Vats et al., 2022). Persistent in Earth’s crust, HMs enter microbial ecosystems both as part of natural and anthropogenic activities (e.g., mining, fuel combustion, industrial emissions, aquaculture, and AG activities) (Koner et al., 2024; Biswas et al., 2021). Long-term geochemical processes, as for example the volcanic eruptions and weathering of basalt bedrock layers, liberates metals like arsenic (As), cadmium (Cd), chromium (Cr), nickel (Ni), lead (Pb), zinc (Zn), Hg, and copper (Cu) into surrounding soils, favoring environmental and health risks (Koner et al., 2024; Biswas et al., 2021). At the same time, human activities, such as the agricultural runoff, discharge of industrial wastes, and sewage sludge application, also contribute to HM accumulation in the natural ecosystems (Biswas et al., 2021). Due to their toxic emissions, Cd, Hg, and Pb are among the HMs with the most significant impact on the natural environment. Data from the European Environment Agency (EEA), encompassing 33 European countries, indicates a decreasing trend in the emissions of these metals since 1990. Due to their toxic emissions, Cd, Hg, and Pb are among the HMs with the most significant impact on the natural environment (Zieliński et al., 2021). Data from the European Environment Agency (EEA), encompassing 33 European countries, indicates a decreasing trend in the emissions of these metals since 1990 (Zieliński et al., 2021). However, some EEA-associated countries continue to face challenges related to high levels of these pollutants. Currently, public energy and heat-generating plants are the largest sources of Hg, Pb, and Cd emissions. Notably, in 2017, Poland led Europe in lead emissions, accounting for approximately 20% of the continent’s total. Additionally, Poland, Germany, and Italy were responsible for the highest levels of cadmium emissions in Europe (Zieliński et al., 2021).

Selectively, the persistence of these metals exerts pressure on microbial communities and facilitates the development of resistance mechanisms that are not only metal-specific but also cross-resistant against antibiotics (Koner et al., 2024; Sodhi et al., 2023; Dickinson et al., 2019). For a long time, the vast diversity of ARGs, within the environmental resistome, was largely overlooked by the scientific community, as studies on antibiotic resistance focused primarily on clinical settings (Vats et al., 2022). However, growing evidence highlights that resistance genes driving MDR (Multi Drug Resistant) continually derive from non-pathogenic environmental microbes in non-clinical environments (Rutgersson et al., 2014; Xiong et al., 2014). A notable example is ARGs coding for resistance to aminoglycosides and vancomycin, which exhibit similar mechanisms of action in both environmental and clinical isolates (Vats et al., 2022). When transferred to human pathogens, these environmental ARGs can evolve into clinically significant resistance determinants under the selective pressures of antibiotic use in human settings (Vats et al., 2022).

Soil microbiomes serve as essential reservoirs of resistance genes, which can be exchanged with pathogenic bacteria through HGT (Nguyen et al., 2023). As, Hg, Cu, Cr, Cd, Zn, and Pb are found in irregular concentrations in animal feed supplements, plant and animal manures (Vats et al., 2022). Among these, Zn and Cu are particularly notable as they are extensively accumulated in animal feed, where they are used as growth promoters due to their antimicrobial properties (Vats et al., 2022). Metal-contaminated soils, in particular, act as selective environments that promote the proliferation of AMR, which is broadly enabled by bacterial efflux pumps that not only expel toxic metal ions but also provide resistance to antibiotics (Nguyen et al., 2023). When present in surplus, HMs such as Cd, Pb, and As can cause significant oxidative damage in bacterial cells, generating free radicals that lead to DNA damage and destabilizing cell membranes through lipid peroxidation (Bazzi et al., 2020). Moreover, HM ions can interfere with enzymatic functions by forming complexes containing enzymes with thiol (R-SH). For instance, metals like Hg^2+^, Ag^+^, and Cd^2+^ can covalently bind to sulfhydryl functional groups (R-SH) within enzymatic active sites, inducing conformational changes that inhibit their functionality (Bazzi et al., 2020). Additionally, HMs can act as competitive inhibitors by displacing essential ions from their binding sites, further disrupting cellular processes. Beyond the direct consequences of HMs and microplastics, dissolved organic matter (DOM) plays another important role in modulating microbial ecosystems and shaping the dynamics of environmental resistance genes. DOM contains a broad range of dissolved organic molecules the environment and represents the most complex molecular mixtures known to date (Zark and Dittmar, 2018).

The DOM indicator is the primary carbon and energy source for microorganisms and, as such, likely has a climactic role in the dynamics of ARGs in the environment (Li et al., 2024). DOM supports the ecological niches for ARG hosts by providing the conditions that support microbial activity and growth (Li et al., 2024). Landfill leachates, with their heightened concentrations of DOM, exemplify how this organic matter interacts with contaminants. Beyond its direct impact on ARGs, DOM significantly influences the environmental behavior of HMs (Li et al., 2024). The bioavailability and toxicity of these metals are often altered by the complex or colloidal mixtures they form with DOM. HMs can be categorized based on their toxicity levels, ranging from less toxic metals like Copper (Cu), and tin (Sn) to highly toxic ones such as Hg, As, Cd, Pb, and vanadium (V), which lack any biological function (Anedda et al., 2023; Balta et al., 2019). HMs are characterized by their high electrical conductivity, malleability, metallic luster, and ability to transfer electrons and form cations, of which Ni and Cr play essential biological roles, serving as cofactors for critical enzymatic activities (Bazzi et al., 2020). Ni is vital for the proper functioning of urease, an enzyme involved in nitrogen metabolism, while Cr is necessary for the activity of cytochrome P450 enzymes, which are key to various metabolic pathways (Bazzi et al., 2020). For instance, Pb exhibits very high adsorption affinity to organic matter, and Cr has high positive correlations with dissolved organic carbon in landfill leachates. Such interactions can enhance the bioavailability of certain HMs, amplifying their correlation with ARG abundance (Li et al., 2024). HMs interact dynamically with DOM (including humic and fulvic acids) that also compete for microplastic adsorption sites (Wu et al., 2024). The complexation between DOM and HMs can either reduce or enhance their adsorption, depending on the nature of the specific interactions. Polypropylene (PP) and polyvinyl chloride (PVC), with larger surface areas and greater porosity, also show higher capacities to adsorb Pb, Cu, and Cd (Wu et al., 2024). Also, pH values dramatically affect the adsorption efficiency of microplastics toward HMs. Reduced competition of hydrogen ions and an increase in negatively charged sites on microplastic surfaces at higher pH values leads to improved adsorption of metal cations (Wu et al., 2024).

In this context, microplastics add another layer of complexity to such diverse environmental conundrum (Wu et al., 2024). Pervasive everywhere in aquatic, terrestrial and atmospheric ecosystems, microplastics are an ideal substratum for microbial colonization, allowing for the establishment of biofilm hotspots in which HGT of resistance genes takes place (Engin et al., 2023; Wu et al., 2024). Meanwhile, HMs, both naturally occurring and anthropogenically introduced, present microbial communities with selective pressures that lead to co-selection of heavy metal resistance genes (HMRGs) and antibiotic resistance genes (ARGs) (Engin et al., 2023; Wu et al., 2024; Hou et al., 2025). The association of these genes mediated through chromosomal mutations on mobile genetic elements (MGEs - like plasmids and integrons), enables propagation of several of these resistance traits and at the same time, increases the ecological and health risks from contaminated environments (Dickinson et al., 2019; Li et al., 2024; Hou et al., 2025).

It has been demonstrated that sub-inhibitory concentrations of HMs, such as Cu^2+^, Ag^+^, Cr^6+^, and Zn^2+^, can enhance the HGT of plasmid-mediated antibiotic resistance among bacteria (Pu et al., 2021). This gene transfer relies on cell-to-cell contact through conjugation and is more likely to occur within the same species but can also extend across genera (Pu et al., 2021). For instance, most transconjugants in studies were identified as *Escherichia hermannii* (*E. hermannii*) and *Shigella boydii* (*S. boydii*), accentuating the ability of HGT to enable distantly related taxa to share genetic material, fostering evolutionary adaptation (Pu et al., 2021). Alarmingly, potential human pathogens were also observed among the transconjugants, including *Klebsiella pneumoniae*, *E. hermannii*, *Acinetobacter tandoii*, and *S. boydii*, associated with severe health issues such as pneumonia, urinary tract infections, and bacillary dysentery (Pu et al., 2021).

Recent studies revealed the profound implications of these interactions, where metals (i.e., As, Cd, Cr, Ni, Pb, Zn, and Hg) have been shown to upregulate efflux pump genes in bacteria, enhancing their ability to resist both metals and antibiotics (Koner et al., 2024; Gupta et al., 2023). Bioavailable forms of HMs also better predict the abundance of ARG compared with total metals concentrations, demonstrating that bioavailability of contaminants is a key driver of resistance dynamics (Li et al., 2024; Hou et al., 2025). Consequently, microplastics not only act as vectors for pollutant transport but also act synchronously, serving as a stable substrate for microbial gene exchange, forming a nexus where pollutants and resistance determinants merge (Nguyen et al., 2023; Komijani et al., 2021).

However, the interplay between DOM and HMs is not always mitigating. Despite the complexation process reducing HMs toxicity, highly bioavailable forms of metals remain strongly linked with ARG proliferation. Among the factors that modulate the evolution of ARGs in DOM-enriched environments are mechanisms such as electrostatic interactions, hydrophobic interactions, hydrogen bonding and the changes in the microbial community structure (Li et al., 2024). Therefore, DOM serves a dual role as a supporter of microbial growth and as a mediator of pollutant behavior—accentuating its binding role in shaping resistance dynamics in contaminated ecosystems (Li et al., 2024).

Microplastics and HMs are ubiquitous in environments such as urban wastewater systems and remote agricultural soils, emphasizing the need for understanding the roles they play in fostering AMR (Komijani et al., 2021). Such pollutants are deposited into ecosystems from the agricultural runoff and industrial discharges, and disrupt native microbial communities, reduce the biodiversity, and give an advantage to resistant pathogenic strains (Koner et al., 2024; Biswas et al., 2021). Furthermore, the presence of HMs (e.g., Cr, Cd, Ni, As, Cu, and Hg) previously correlated with high abundance of ARG (e.g., *aad*A - aminoglycoside-3″-adenylyltransferases, *bla*CTX-M—*β*-lactamases, *bla*SHV—ampicillin resistance, *erm*B—erythromycin resistance, *mef*A—erythromycin resistance, *tet*M—tetracycline resistance, *tet*Q—tetracycline resistance efflux pump, *sul*-1 and *sul*-2—sulphonamide resistance) in landfill leachates interacting with dissolved organic matter, complicates the environmental behavior of these contaminants by means determining their bioavailability and toxicity (Sodhi et al., 2023; Li et al., 2024; Hou et al., 2025; Shen et al., 2023). A recent analysis of metagenome-assembled genomes (MAGs) revealed 1,145 MAGs with 29 bacterial phyla that harbored ARGs and MRGs simultaneously (Hou et al., 2025). Among these, bacteria from the phylum *Pseudomonadota* were identified as the carriers of ARG-MRG combinations. Notably, this phylum exhibited high co-occurrence of specific ARG-MRG pairings, including β-lactam-Pb, multidrug-As, polymyxin-Cd and quinolone-Cd, suggesting a coexistence and potential dissemination of resistance traits across environmental microbial communities (Hou et al., 2025).

This review seeks to clarify the complex interplay between microplastics, HMs and AMR, with a focus on the mechanisms driving the uptake and propagation of resistance. The environmental dimensions of this phenomenon must be considered as they become more relevant in the context of the intensifying general crisis of AMR. Therefore, this review seeks to bridge this knowledge gap in pollutant-driven resistance mechanisms to contribute to the development of integrative solutions in the future that balance environmental and antimicrobial stewardship to protect ecosystem health and human well-being.

## 2 Emerging pollutants: microplastics and antimicrobial resistance

Microplastics have been identified as hubs that enrich antibiotic-resistant bacteria and pathogens in municipal activated sludge and other aquatic and terrestrial environments (Buelow et al., 2021). Recent studies assessing the impact of plastic mulch in agriculture have revealed additional potential drawbacks, such as reduced crop yields due to high levels of plastic residue in fields, an increased abundance of mycotoxigenic fungi, and elevated levels of the mycotoxin deoxynivalenol (Micallef et al., 2023). Microplastics have been shown to accumulate pollutants and support biofilms that are enriched with ARGs in bacterial pathogens. In natural environments, biofilms represent the preferred lifestyle of bacteria, supporting high bacterial densities, facilitating gene transfer, and becoming enriched with ARGs in polluted environments. These biofilms provide optimal conditions for the transfer and evolution of ARGs within bacterial populations (Buelow et al., 2021).

In this context, microplastics are conventional plastic particles with dimensions smaller than 5 mm. Microplastics have been proposed as hotspots for horizontal gene transfer in water sources and are relatively new factors in the evolution of environmental microbial communities (Magnano San Lio et al., 2023). Consequently, metagenomic sequencing methods could be employed to assess the impacts of microplastics on the spread of ARGs in various environmental settings, including water, soil, and air (Magnano San Lio et al., 2023). Mixed pollutants adhered to microplastics and produce favorable conditions for co-selection, especially in landfill leachate, which is known to contain free DNA, a significant source of resistance genes in microplastics (Zhang et al., 2024). The co-occurrence of disinfectants, metals and antibiotics on microplastics exerts environmental pressure on microbes, leading to the generation and spread of multi-resistant bacteria (Zhang et al., 2024).

Several critical food safety issues at the farm level persist in low- and middle-income countries (Seyoum et al., 2024). These issues include the lack of veterinary services for sick animals, poor adherence to good farming practices (such as inadequate hygiene), and inappropriate farm equipment, such as milk plastic containers, contributing to foodborne pathogen contamination (Seyoum et al., 2024). Plastics or microplastics pollution impose severe consequences, through their ability to act as a “Trojan horse,” adsorbing chemicals and microorganisms from the surrounding environment via biofilm formation (Tuvo et al., 2023). This biofilm ecosystem formed on plastic debris is often called the “plastisphere” (Tavelli et al., 2022). Bacteria present in this plastisphere are embedded in a self-secreted exopolymeric substance (EPS), which can have various functions and structures (Tavelli et al., 2022). For example, in the EPS of the food pathogen *Salmonella enterica*, cellulose and O-antigens have been identified as governing components for facilitating attachment and biofilm formation, and also for ensuring biofilm persistence on plastic surfaces (Tavelli et al., 2022). The microbial composition of the plastisphere depends on numerous factors, including geographical location, season, and the type of polymer (Tavelli et al., 2022). The composition of the plastisphere is also affected by geographical and seasonal factors (e.g., polypropylene, polyvinyl chloride and high-density polyethene) impacting on the bacterial composition and specifically on the presence of foodborne pathogenic bacteria such as *L. monocytogenes*, *E. coli*, and *Enterobacter* spp. and viruses (Witsø et al., 2023). The diversity and taxa of the plastispheres are also influenced by the time plastics spent in the river, the season and the geographical location. The bacterial diversity in the plastisphere varied significantly between June and September, with generally higher diversity observed during June (Witsø et al., 2023).

The risk associated with food-borne exposure to microplastics for human health is a pressing concern. The particles themselves present several hazards such as a physical hazard due to their size, a chemical hazard from unbound monomers, sorbed chemicals, additives from the environment, and a biological threat from the microorganisms that may bind and colonize on microplastics (biofilms) (Tuvo et al., 2023). The spread of ARGs and micro/nano plastics in soil environments exacerbates the migration of ARGs into the food chain, increasing the concerns related to their probable threat to human health via food consumption (Tuvo et al., 2023).

Soil has unpredictably transformed into a powerful microplastic reservoir as increasing amounts enter soil ecosystems (Zhu et al., 2023). These plastics create a new niche for soil microbes, allowing a diverse range of microorganisms to colonize the plastic surface to create a “plastisphere” (Zhu et al., 2023). A plastisphere can entrap numerous foodborne pathogens, including species of our interest such as *Campylobacter* spp., *E. coli*, *Vibrio* spp., *C. perfringens*, *B. cereus*, and others (Tavelli et al., 2022). Data has shown that the plastisphere contains more potential human pathogens, such as *P. aeruginosa* and *Vibrio fluvialis*, than the surrounding soil (Zhu et al., 2023). Moreover, the plastisphere acts as a hotspot for HGT, potentially accelerating the transfer of genes encoding virulence factors and AMR (Zhu et al., 2023). Therefore, the plastisphere community is a novel vector for transmitting human pathogens, especially if the plastic is exposed to fecal contamination from sources such as wastewater, organic manures and livestock feces (Quilliam et al., 2023).

The abundance of potential human pathogens in the plastisphere rises greatly with soil moisture. Of late, the *Vibrio* genus, which includes several fish and human pathogens, has been identified as an early colonizer of microplastics (Tavelli et al., 2022), acting as a pioneer by commencing the colonization of microplastics and promoting the adhesion of other bacterial species. In addition to being key players in early microplastic colonization, *Vibrio* spp. are repeatedly primary components of the plastisphere community (Tavelli et al., 2022). Specifically, *V. parahaemolyticus*, *V. cholerae*, and *V. vulnificus* have been found on the surfaces of predominantly polyethylene and polypropylene particles (Tavelli et al., 2022). Meanwhile, *Campylobacter* spp. and *Pseudomonas* spp. have been found to prevail mostly on polyethylene microplastics. While the pathogenicity of *Campylobacter* spp. is well-documented as a foodborne agent, *P. aeruginosa* may also play an underestimated role in food safety (Tavelli et al., 2022).

## 3 Heavy metals as drivers of antimicrobial resistance

As our climate warms, the concentrations of heavy metals in soil and water, and the possibility of increased bacterial absorption is more probable and can trigger AMR through co-resistance mechanisms (Magnano San Lio et al., 2023). Heavy metals are also widely prevalent in the environment due to anthropogenic actions such as mining, industrial processes, and agricultural practices (Buelow et al., 2021). These metals often originate from historical industrial pollution and tend to bioaccumulate in lakes, riverbeds, and estuaries, especially in industrial areas which are enriched with heavy metals or PTEs (potentially toxic elements) (Buelow et al., 2021).

The correlation between metal contamination and AR proliferation was initially observed in heavily polluted areas (Knapp et al., 2021). These include the outflows of inadequately treated wastewater and biosolids, agricultural waste sites, industrially contaminated regions, and through direct exposure experiments (Knapp et al., 2021). The presence of AR in these scenarios often indicates exposure to high levels of metal pollution. Heavy metal accumulation and environmental spread are associated with industrial pollution (Knapp et al., 2021). In the environment, metals or PTEs can play a dual role in microbial cellular health (Knapp et al., 2021). As micronutrients, they contribute to essential biological functions, but at elevated concentrations, they induce stress responses and foster the development of resistance mechanisms. Copper, commonly present in animal feed, along with pollutants like arsenic and mercury that infiltrate the food chain, can facilitate the co-selection of ARG (Rebelo et al., 2023). This occurs because these elements often share genetic contexts for metal tolerance genes and ARGs on various mobile genetic elements (Rebelo et al., 2023). A notable example of this is the adaptation of bacteria to high copper levels in metal-rich soils, where they develop specific copper-resistance mechanisms (Knapp et al., 2021). Contrariwise, metals like chromium and mercury offer minimal biochemical benefits and primarily cause oxidative stress (Knapp et al., 2021). A concerning aspect is that genes conferring metal resistance are often located near ARGs on mobile genetic elements, facilitating the co-selection and spread of AMR (Knapp et al., 2021). The co-selection of metal resistance and AR (or other resistance mechanisms against toxic organics or disinfectants) can occur through two primary processes: co-resistance and cross-resistance (Knapp et al., 2021). Co-resistance happens when the selection of one resistance phenotype simultaneously selects for other genes located on the same genetic element (Knapp et al., 2021). Conversely, cross-resistance occurs when both the antibiotic and the metal share similar biochemical pathways or cellular targets (Knapp et al., 2021). In this case, the resistance response triggered by one agent provides defense against both the metal and the antibiotic. Thus, the environment serves as both a reservoir of resistance traits and a bioreactor with chemical stressors, fostering opportunities for genetic exchange (Knapp et al., 2021).

High temperatures are not only linked to heavy metal accumulation in crops but also the chemical contamination of crops (Yeni and Alpas, 2017). For instance, drought combined with high temperatures can lead to wildfires, and incomplete combustion during these fires might generate organic pollutants that contaminate food products via depositing into water or soil. Alongside metals, certain persistent organic compounds, such as pesticides, polychlorinated biphenyls (PCBs), and disinfectants, co-select for antimicrobial (including antibiotic) resistance genes (Knapp et al., 2021). Additionally, polycyclic aromatic hydrocarbons (PAHs), which are widespread environmental pollutants, have the ability to increase the abundance of AMR in microbial communities (Knapp et al., 2021). Climate change-induced alterations in microbial community composition may bring previously disparate bacterial species closer to humans and animals (Knapp et al., 2021). This increased proximity can directly enhance levels of HGT, which has been linked to the causation and dispersion of ARGs in the environment.

## 4 Implication of HMs and antibiotics in bacterial survival and dissemination of AMR

### 4.1 Co-resistance and cross-resistance

The co-occurrence of heavy metal resistance and AMR is primarily governed by two mechanisms such as co-resistance and cross-resistance (Zieliński et al., 2021; Anedda et al., 2023; Wu et al., 2024). In co-resistance, MRGs and ARGs are located on the same MGE (mobile genetic elements), such as plasmids, transposons, integrative-conjugative elements or integrons (Zieliński et al., 2021; Rilstone et al., 2021). This physical linkage facilitates their simultaneous transfer between bacterial populations, amplifying the spread of resistance traits. In *Serratia marcescens*, resistance to antibiotics such as chloramphenicol, kanamycin, and tetracycline is genetically linked to resistance genes for HMs, including As and Hg, suggesting a shared genetic basis for multi-resistance traits (Bazzi et al., 2020). Similarly, whole-genome sequencing analyses of *Salmonella typhi* have uncovered genetic associations between Hg resistance and resistance to a broad spectrum of unrelated antimicrobial agents, including ampicillin, chloramphenicol, streptomycin, sulfonamide, and trimethoprim located on a conjugative plasmid named pHCM1 (Vats et al., 2022; Bazzi et al., 2020). Hg resistance genes were previously genetically linked to ARGs on plasmids, allowing co-transfer during bacterial conjugation between Enterobacteriaceae and recipient strains (Vats et al., 2022). Earlier findings demonstrated this by identifying plasmids in *Salmonella abortus* that conferred co-resistance to ampicillin and As, Cd, Hg, and Cr (Vats et al., 2022; Rilstone et al., 2021; Ghosh et al., 2000). Removal of these collective plasmids resulted in strains sensitive to antibiotics and HMs, confirming that the resistance genes were carried on plasmids. In contrast, *Enterococcus faecalis* continued to exhibit resistance to amikacin, aztreonam, gentamicin, and Pb2+ even after plasmid removal (Rilstone et al., 2021).

For instance, the tetracycline resistance gene *tet*A has been positively linked with the Cu resistance gene *cop*B and the Hg resistance gene *mer*R, illustrating the influence of HMs on the prevalence of ARGs (Zou et al., 2021). Further studies demonstrated that when *E. coli* DH5α acquired plasmids from *B. megaterium* and *Shewanella oneidensis*, the resulting strains resisted metals and antibiotics, showcasing the genetic connectivity between the metal and AMR (Xu et al., 2017). Others identified significant positive correlations between Ni and *tet*O and between Zn, Cu and *sul*1 (Yan et al., 2020). Equally, Cd and Cr were negatively correlated with *sul*1. The study also revealed that HM-resistant bacterial phyla, such as Actinobacteria and Proteobacteria, play a pivotal role in disseminating ARGs through co-resistance and cross-resistance mechanisms.

Cross-resistance, on the other hand, arises when a single resistance mechanism, such as a multi-drug efflux pump, can protect bacteria from both HMs and antibiotics by actively expelling these compounds out of the cell (Zieliński et al., 2021; Anedda et al., 2023). In *Listeria monocytogenes*, the MdrL efflux pump confers resistance to various substances, including Cr, Co, and Zn, as well as antibiotics such as clindamycin, erythromycin, and josamycin (Bazzi et al., 2020). Again, the DsbA-DsbB (disulfide bond) efflux system in *Burkholderia cepacia* mediates cross-resistance to *β*-lactams, erythromycin, kanamycin, novobiocin, and ofloxacin, as well as the metal ions Zn^2+^ and Cd^2+^ (Bazzi et al., 2020). Furthermore, in *Campylobacter jejuni*, resistance to antimicrobial agents alongside Co and Cu is linked to the activity of the CmeABC efflux pump (Bazzi et al., 2020; Lin et al., 2002). Bacteria isolated from HM-contaminated mining sites often develop antibiotic resistance, as previously shown in *Burkholderia dabaoshanensis*, showed drastic Cd tolerance with a minimum inhibitory concentration (MIC) of 22 mmol/L (Zou et al., 2021). Additionally, resistance testing indicated this strain can thrive in the presence of ampicillin, kanamycin, and streptomycin. A recent study highlighted that metals significantly impact the ARG profile more than antibiotics themselves (Mazhar et al., 2021). The *cnr* (nickel-cobalt resistance) and *czc* (cobalt-zinc-cadmium resistance) determinants showed substantial homology in their encoded structural proteins and the cations they potentially export. Furthermore, certain metals, such as As, Zn, Mb, Co, and Ag, as well as antibiotics (i.e., ciprofloxacin, tetracycline, chloramphenicol, and beta-lactams), share structural and functional similarities (Mazhar et al., 2021). These similarities often involve mechanisms such as reduced membrane permeability, which facilitates cross-resistance. It has been reported that ARGs were found to cluster significantly with Cd, while Zn was present in high abundance, indicating shared resistance determinants between these two HMs that were strongly correlated with ARGs, particularly with *tet* genes, proposing that these genes were enriched through co-selection (Mazhar et al., 2021).

### 4.2 Co-regulation, co-selection, and biofilm formation

Bacterial biofilm formation is a critical stage in the bacterial life-cycle being co-regulated along with virulence and antibiotic resistance (Sinha et al., 2022). For example, a potential mechanism could include horizontal gene transfer which occurs faster within biofilms leading to the development of MDR bacteria following transfer of ARGs (Mendes et al., 2023). Co-regulation and biofilm formation amplify the co-selection of AMR and heavy metal resistance (Zieliński et al., 2021; Anedda et al., 2023). This interconnected regulation enables the expression of resistance genes in response to exposure to a single agent (Wu et al., 2024). Metal exposure has been shown to upregulate the expression of ARGs, enhancing bacterial survival under antibiotic stress (Vats et al., 2022). For instance, exposure to HMs can activate the *czc* operon, which encodes a metal efflux system designed to expel toxic metal ions (Wu et al., 2024; Perron et al., 2004). Remarkably, this activation can simultaneously induce the expression of the *mex* operon, which confers resistance to specific antibiotics (Wu et al., 2024; Perron et al., 2004). Such co-regulation highlights the intricate molecular interplay that allows bacteria to adapt and survive in environments containing diverse stressors, contributing significantly to the co-selection of AMR (Wu et al., 2024). Co-regulation, the least common mechanism of co-selection, occurs when a shared regulatory protein controls resistance genes for antimicrobial agents and HMs (Bazzi et al., 2020; Pal et al., 2017). A well-studied previous example of this mechanism is the CzcS-CzcR dual-component regulatory system in *Pseudomonas aeruginosa* (Perron et al., 2004). This system confers resistance to Zn^2+^, Cd^2+^, and Co^2+^ by activating the expression of the czcCBA efflux pump. At the same time, it contributes to resistance against the carbapenem antibiotic imipenem by repressing the expression of the *opr*D porin-encoding gene, which reduces the antibiotic’s uptake into the bacterial cell (Perron et al., 2004). In the Enterobacteriaceae bacteria LSJC7, exposure to arsenate significantly increases the expression of multiple ARGs, including *emr*D and the tetracycline resistance gene *tet*34 (Chen et al., 2015). This effect occurred even at environmentally relevant concentrations of arsenate, indicating the potential ecological risks associated with HMs contamination. Likewise, oxidative stress caused by ions such as Cd^2+^, Cu^2+^ and dichromate (Cr₂O₇^2−^) upregulates the expression of the universal response regulator protein *Sox*S in *E. coli* and *Salmonella* spp. (Vats et al., 2022; Kaur et al., 2021). *Sox*S, in turn, activates the multidrug efflux pump *Acr*AB, which conferred resistance to a wide range of antibiotics. Additionally, Cd^2+^ and Cu^2+^ ions served as inducers that derepress the *Mar*RAB regulatory operon, a key player in multiple antibiotic resistance, in *E. coli* and *Salmonella* (Vats et al., 2022).

Co-selection of metal and antibiotic resistance arises from similar resistance mechanisms. Co-selective pressure can harbor AMR in microbial communities, even without antibiotics (Arya et al., 2021). The first case of heavy metal resistance in bacteria was recorded by Moore in 1960, who found Hg-resistant *Staphylococcus aureus* during a hospital outbreak (Moore, 1960). Richmond and John later in 1964 noted the genetic link between mercury resistance and high penicillinase activity in *S. aureus* isolated from wounds (Richmond and John, 1964). Timoney and collaborators in 1978 discovered *Bacillus* strains in sewage sludge with combined Hg and ampicillin resistance, indicating that sludge environments could select for both resistance types simultaneously (Timoney et al., 1978). Research on pathogens like *S. aureus* and *E. coli* shows that plasmids with ARGs often carry MRGs, highlighting the potential for simultaneous selection and spread of these traits due to overlapping selective pressures (Vats et al., 2022; Hobman and Crossman, 2015). Sequenced bacterial genomes and plasmids show a high prevalence of plasmids in significant genera like *Escherichia*, *Klebsiella*, *Salmonella*, and *Staphylococcus* (Vats et al., 2022). Many plasmids contained antibiotic and HM resistance genes, accentuating the potential for co-selection via co-resistance. This indicates the pervasive impact of HM-induced antibiotic resistance in clinical pathogens, a largely unexplored area. A study on *Salmonella enterica* serovar Typhi confirmed the connection between metal and antibiotic resistance (Kaur et al., 2018). Cadmium supplementation made sensitive isolates resistant and raised the MICs of antibiotics—ampicillin, ciprofloxacin, chloramphenicol, and ceftizoxime—in resistant isolates. This effect resulted from a co-regulatory mechanism of co-selection, emphasizing the role of heavy metals in exacerbating AMR in clinical pathogens.

Biofilms are complex microbial multispecies communities covered in a protective extracellular nutritive matrix providing a survival advantage under stress conditions, including HMs exposure. The biofilm matrix can bind HM ions, preventing their diffusion and reducing their toxicity, therefore promoting genetic mutations and the persistence of ARGs. While co-regulation occurs when the presence of HMs induces changes in ARG expression, further promoting AMR dissemination (Anedda et al., 2023; Wu et al., 2024). The propagation and persistence of AMR in primary food production system is strongly linked to HMs present in the environment. Their influence goes through several transmission routes such as through soil, water, and food, and affecting plants, animals and humans (Anedda et al., 2023). Young monoculture pathogenic biofilms (*P. aeruginosa*, *E. coli*, and *S. aureus*) cultured for ~2–6 h exhibited ~2–600 times more resistance to Pb^2+^ and Cu^2+^ than their planktonic counterparts (Rilstone et al., 2021). However, this advantage was reported to decline after 24 h, likely due to increased energy demands of resistance mechanisms like efflux pumps during exponential growth compared to the stationary phase.

For example, iron oxides are vital for metal adsorption in drinking water distribution systems (DWDSs) (Rilstone et al., 2021). They absorb arsenic (As^3+^ and As^5+^) in a specific form and bind Pb^2+^ at the interface of *Burkholderia cepacia* biofilms and hematite (Fe^3+^ oxide), initiating complex biomineral formation. Similarly, biogenic Mn^2+^ oxides select Pb^2+^ in *Leptothrix discophora* biofilms and Zn^2+^ in *Pseudomonas putida* biofilms. While Ni^2+^ selectively adheres to silicate-rich sheaths, showing no significant binding to biofilm bacteria or extracellular substances (Rilstone et al., 2021). In terms of AMR in DWDS biofilms, arsenic-induced ARB can persist up to 4 days without antibiotics or metals, raising concerns about ARB and ARG spread in drinking water (Zhang et al., 2020). Pathogenic genera such as *Escherichia*, *Klebsiella*, *Shigella*, and *Salmonella*, pose significant health risks if AMR develops within them (Rilstone et al., 2021; Li et al., 2017). The five most common ARG types in DWDS biofilms confer resistance to *β*-lactams, macrolide-lincosamide-streptogramins, bacitracins, aminoglycosides, and tetracyclines (Li et al., 2017). Of the 23 identified MRG types, the prevalent ones provide resistance to Zn, Cu, Ni, Mn, and Hg (Li et al., 2017).

Metals exert their toxicity on bacteria via several recently described mechanisms, including the inactivation of proteins and enzymes by inappropriate binding sites, the generation ROS, and interference with nutrient uptake and cellular structures (Anedda et al., 2023). To counteract these toxic effects, bacteria have developed sophisticated resistance mechanisms via the sequestration of toxic metals through complex formation, the detoxification of intracellular ions through reduction processes, and the active extrusion of toxic ions utilizing the efflux systems. These adaptations are mediated by HMRGs, which are present both in the core genomes of bacteria and on MGEs (Anedda et al., 2023).

Efflux pumps are bacteria’s primary mechanisms to resist heavy metal toxicity, extruding metal ions via ATP hydrolysis or proton gradients (Bazzi et al., 2020). There are five major efflux pump families: ATP Binding Cassette (ABC) family expels metal ions using ATP; Resistance-Nodulation-Cell Division (RND) family pumps out cations and antibiotics with proton motive force; Small Multi-Drug Resistance (SMR) family operates like RND, using chemiosmosis; Multi-Drug and Toxic Compound Efflux (MATE) transporters expel various substrates; and Major Facilitator Superfamily (MFS) utilizes proton gradients for ion removal (Bazzi et al., 2020). Basal activity alone does not confer significant resistance, but mutations in promoter regions or changes in regulators can enhance pump overexpression, increasing resistance (Bazzi et al., 2020). ABC transporters and RND/SMR families are vital in bacterial defense, emphasizing their role in resistance trait spread. The whole-genome analysis of *Pseudomonas putida* KT2440 earlier revealed the presence of an RND-type efflux pump, which confers resistance to tetracycline, Rb^+^, and Cr₂O₇^2−^, suggesting the versatility of RND-type efflux systems in mediating resistance to both antibiotics and HMs (Rilstone et al., 2021).

The subsequent resistance mechanism is the intracellular sequestration of metal ions, a bacterial resistance mechanism that involves binding these ions to specific metal-binding proteins, including metallothioneins, glutathione, and metallochaperones (Bazzi et al., 2020; Ianeva, 2009). Metallothioneins, which are cysteine-rich polypeptides, effectively trap toxic heavy metals such as Cd, Hg, Pb, and Zn, enabling bacteria to tolerate high concentrations of these metals. For instance, *Synechococcus*, *Pseudomonas*, and *Anabaena* spp. rely on metallothioneins to survive in HM-contaminated environments (Bazzi et al., 2020; Naik et al., 2012). Conversely, glutathione functions as an alternative chelator that scavenges and detoxifies heavy metals through its thiol (-SH) group (Bazzi et al., 2020). This mechanism plays a significant role in Cd tolerance, as observed in *Rhizobium leguminosarum*, where GSH binds to and neutralizes Cd ions, reducing their toxic effects on cellular components (Lima e Silva et al., 2012). Metallochaperones, a class of proteins that assist in the transport of metal ions within cells, further enhance bacterial metal tolerance by binding, transporting, and delivering metal ions to metalloenzymes, thereby minimizing their poisonous impact on cellular compartments. Copper-binding metallochaperones such as CusF and periplasmic chaperones like PcoC and PcoE protect bacterial cells by managing copper ions in both their Cu^1+^ and Cu^2+^ forms (Bazzi et al., 2020; Pal et al., 2017). This targeted sequestration ensures proper metal homeostasis within the cell while preventing oxidative damage. In addition to protein-based mechanisms, some bacteria sequester heavy metals through precipitation. For example, *Bacillus* spp., *Staphylococcus aureus*, *Shewanella* spp., *Providencia* spp., and *Vibrio harveyi* precipitate Pb as phosphate salts, while Ni complexes with phosphate ions (PO₄^3−^) form intracellular precipitates (Bazzi et al., 2020). These combined strategies allow bacteria to adapt to and survive in environments contaminated with HMs, effectively neutralizing their toxicity and maintaining cellular function.

### 4.3 HMs pollution and its role in AMR spread across soil and water systems

As discussed, the presence of HMs (Pb, Hg, As, Cr, Cd, Ni) and other elements in soil and water environments plays a crucial position in shaping microbial community dynamics and the distribution of ARGs as well as MRGs via complex ecological pressures. These pressures can co-select microbial populations with ARGs and MRGs, accelerating AMR spread through HGT and genetic exchanges. Soil and water environments, including agricultural soils, serpentine ecosystems, rivers, and landfill leachates, act as reservoirs and pathways for HMs transmission (Koner et al., 2024; Li et al., 2024; Gupta et al., 2022).

#### 4.3.1 Soil environments

According to the recent soil studies, it was highlighted that Cr and Ni contamination often induces resistance mechanisms, particularly tetracycline resistance (e.g., *tet*M, *tet*A) (Koner et al., 2024; Li et al., 2024; Gupta et al., 2022). The formation of metal complexes, like insoluble Cd-sulfides, can decrease toxicity, yet persistent low bioavailability continues to exert selective pressure (Li et al., 2024; Zhao et al., 2020). In neutral soils, As and especially Cr have been shown to exert a stronger influence on bacterial community (*Acidothermus*, *Acinetobacter*, *Anaeromyxobacter*, *Delftia*, *Citrobacter*, and *Stenotrophomonas*) structure and ARG abundance (Ma et al., 2020). Metals like As, Sb, Cd, and Zn positively correlate with bacterial genera like *Arthrobacter*, *Gaiella*, and *Sphingomonas* (Ma et al., 2020).

In acidic soils, As, Cd, and Zn have a more pronounced mobility effect correlating positively with bacterial genera such as *Streptomyces*, *Arthrobacter*, *Flavobacterium*, and others (Ma et al., 2020). Cr shows negative correlations, inhibiting specific bacterial populations, yet some bacteria (e.g., *Lamia*, *Acidothermus*, *Lysobacter*, etc.) thrive under Cr stress. In landfill environments, metals such as Pb, Cr, and Cd frequently co-occur and form complexes with organic matter, colloids, and other soil constituents (Li et al., 2024). These relationships are crucial because metal speciation and bioavailability influence microbial responses (Gadd, 2004). For instance, Cd, Zn, Cu, and Pb can precipitate with sulfides under anaerobic conditions, reducing their solubility in certain settings, while As can exist in more mobile and toxic forms under similar redox conditions (Li et al., 2024; Zhao et al., 2020). A lower soil pH impacts Cd and Pb bioavailability, which increases with organic matter presence, often leading to the co-selection of ARGs, such as *sul*I and *tet*M (Li et al., 2024; Gupta et al., 2022). In serpentine soils, Cr, Ni, and Co favored the proliferation of ARB (e.g., *Pseudomonas*, *Staphylococcus*, *Rubrobacter*) having strong vancomycin and *β*-lactamase resistance genes, i.e., *aac*A, *amp*C, *aph*, *hyg*, *ox*A, *otr*(C), *pen*P, *str*B, *tet*(A/B), *tet*(M/O/Q), *van*(A/B/D) and *van*J dominate, driven by elevated HM stress (Koner et al., 2024). Similarly, *Firmicutes*, *Actinobacteriota*, and certain *Proteobacteria* have also been identified as prominent groups thriving in native microbiomes such as serpentine soils.

#### 4.3.2 Water environments

Water systems, such as rivers and landfill leachates, act as dynamic interfaces where transition metals interact with microbial communities and resistance genes. For example, raised Pb, Cd, Cr, and Ni levels in polluted rivers in the UK and India have significantly increased MRGs and ARGs (e.g., carbapenem, tetracycline, erythromycin) (Gupta et al., 2022). Network analyses demonstrate strong correlations between czcA, rcnA, (Cd, Ni, Co, Zn resilience) and beta-lactamase resistance genes, illustrating co-selection via integrons such as int1 (Gupta et al., 2022). Riverine communities impacted by elevated Ni, Pb, or Cd also display shifts favoring bacteria with enhanced resistance capabilities (Zhao et al., 2020). As and Cd exert co-selective pressures in wetland soils, altering microbial community diversity and leading to a proliferation of ARGs. Similarly, in landfill leachates, the abundance and diversity of ARGs correlate positively with different HMs, suggesting that these pollutants serve as key drivers of co-selection. In anaerobic environments, sulfur-reducing bacteria precipitate metals (e.g., Pb, As, Cd) as insoluble sulfides (Li et al., 2024; Zhao et al., 2020). However, leachate remains a hotspot for ARG diffusion, with *sul*I and *erm*B positively associated with Cr, Pb, and Cd concentrations (Li et al., 2024; Gupta et al., 2022). Experimental evidence shows that Cd addition promoted MDR and HMRGs in water, particularly in opportunistic human pathogens (Vats et al., 2022). Similarly, subinhibitory concentrations of Cr and Cu promote ARG transfer across genera through conjugation (Vats et al., 2022). A multivariate statistical analysis conducted by Ram and Kumar from Indian river, lakes and sewage where *E. coli* isolates revealed that resistance to streptomycin, kanamycin, and tetracycline was strongly correlated with electrical conductivity, finer-sized microplastics, Ni and Mn (Ram and Kumar, 2020). In contrast, seasonal temperature variations significantly influence fluoroquinolone resistance (Levofloxacin, Ciprofloxacin, and Norfloxacin). Larger-sized microplastics cluster with salinity, oxidation–reduction potential (ORP), and Pb. Fecal contamination and ARB emerged to share common sources and processes but showed strong correlations only in river samples (Ram and Kumar, 2020). This discrepancy was explained to be caused due to the dynamic river–human interface, substantial wastewater discharge, stagnant water flow, and urbanization impacts, which differ remarkably from upstream conditions.

### 4.4 Co-selection evidence from environmental studies

Multiple studies, discussing the co-selection mechanism, have shown that exposure to HMs can occur indirectly ARB, even without the presence of antibiotic compounds. This process is facilitated by mobile genetic elements (MGEs), such as integrons (notably intI1) and plasmids, which carry both MRGs and ARGs (Fu et al., 2023). Genes codifying resistance to Co, Ni (e.g., *rcn*A), and Co, Zn, Cd (*czc*A) have been frequently identified as dominant in metal-polluted samples, often co-occurring with ARGs conferring resistance to beta-lactams (e.g., *bla*CTX-M, *bla*NDM-1), macrolides (*erm*A, *mef*A, *mph*A), tetracyclines (*tet*A, *tet*M, *tet*W), and other antibiotics (Gupta et al., 2022). The presence of integrons in metal-rich environments further enhances the likelihood of ARG spread, as integrons facilitate gene cassette integration and mobilization (Koner et al., 2024; Gupta et al., 2022). Similarly, proteins like TetL facilitate tetracycline and Co resistance, illustrating the interconnected selection pressures (Koner et al., 2024). This integron-mediated co-transmission underlines the importance of considering HM pollution as a consequential factor in ARG dissemination. Correlation analyses and multivariate statistical models consistently show strong positive relationships (*r* > 0.80, *p* < 0.05) between HMs and the abundance of ARGs, MRGs, and integrons (Gupta et al., 2022). For example, in polluted riverine systems, bioavailable Cr, Co, and Ni correlate with tetracycline and chloramphenicol resistance genes, while total-Cu and total-Cr correlate with macrolide resistance determinants, confirming the multifaceted nature of co-selection in natural environments. Under anaerobic conditions, toxic As^3+^ promotes ARG co-selection in paddy soils, while Cd solubility can enhance ARG dissemination in landfills (Zhang et al., 2020; Zhao et al., 2020).

### 4.5 Human activities and implications

Human activities, including wastewater discharge, industrial runoff, and extensive fertilizer use, continuously introduce Pb, As, Cd, Ni, and Cr into soils and waters (Zhao et al., 2020; Ram and Kumar, 2020; Peng et al., 2022) and they are most associated with toxicity (Shin et al., 2013). Agricultural soils receiving fertilizer may accumulate HMs that enhance ARG prevalence. Similarly, sewage effluent and landfill leachates serve as hotspots for metal–organic complexes and nutrient-rich conditions that support microbial growth and ARG proliferation (Bazzi et al., 2020; Gupta et al., 2022; Peng et al., 2022). The co-occurrence of metals like Pb, Zn, and Cu with sulfonamide resistance genes (*sul*I, *sul*II) and macrolide resistance genes (*mef*A, *erm*A, *erm*B) in both refuse and leachate demonstrates that these environments are prime grounds for ARG enrichment (Li et al., 2024; Gupta et al., 2022).

In river ecosystems, Ni, Co, Zn, Cd, Cr, and Pb correlate with the abundance of MRGs and integrons, reinforcing the notion that HMs influence the HGT of ARGs. Seasonal and hydrological conditions and varying pH and redox states can further modulate these interactions (Ma et al., 2020). For instance, lower pH conditions increase the solubility and bioavailability of Cd, Pb, Zn, which may intensify selective pressure and promote HMRGs and MDR, respectively.

#### 4.5.1 Lead (Pb)

Lead (Pb) is a heavy metal with a total estimated abundance in earth’s crust of ~0.0013%, with a dense and pliable soft texture, with a silver-bluish hue, which has been and continues to play an integral part in human industrial and technological development (Sevak et al., 2021; Pandey, 2012). Pb is predominantly in two oxidative states, Pb^2+^ and Pb^4+^, where the contamination in the environment is mainly attributed to human activities such as mining, smelting, battery production, paint manufacturing, and the combustion of fossil fuels (Sevak et al., 2021). Furthermore, previous presence of Pb in toys, medicines, explosives, jewelry, paper, metallurgy, automobile sectors have been major contributors to its pollution (Bazzi et al., 2020; Sevak et al., 2021). Despite its low geochemical mobility, anthropogenic activities have propelled Pb to toxicologically relevant concentrations, with 98% of environmental Pb pollution stemming from human enterprises (Sevak et al., 2021). Pb contamination comes mainly from industries such as Pb-acid battery manufacturing, which uses approximately 85% of the world’s Pb (Zhang et al., 2024).

Pb pollution is not specific only to industrial zones as it can be spread and influence air, soil and water quality (Ma et al., 2020). Acidic waters can leach Pb out of plastic PVC pipes into the drink water supply, accompanied by natural processes which can erode Pb into the atmosphere and sediments (Ma et al., 2020; Sevak et al., 2021). Pb also forms compounds [PbCO₃, Pb₂O, Pb(OH)₂, and PbSO₄] which can precipitate in surface and groundwater and absorb onto mineral surfaces (Sevak et al., 2021). As a result, the concentration and bioavailability of Pb in these water systems are affected by various factors, including dissolved salts, mineral composition, and pH levels. Pb is a persistent, non-degradable HM that can traverse distant ecosystems. Of particular concern is the widespread Pb contamination in agricultural regions worldwide, where it can enter the food chain, jeopardizing global food security and public health (Zhang et al., 2024). According to estimates, more than a billion people globally are exposed to hazardous levels of Pb, resulting in intellectual disabilities for over 600,000 children per year (Zhang et al., 2024). The cumulative effects of Pb toxicity highlight significant long-term public health risks, as it can remain in bones for decades and later re-enter the bloodstream, nevertheless was reported to be excreted via feces and urine (Vats et al., 2022; Sevak et al., 2021). Pb is toxic at multiple levels on a cellular basis, known to induce oxidative stress by yielding ROS that damage DNA, proteins and enzymes and interfere with calcium metabolism, leading to metabolic processes compromising with systemic tissue destruction (Bazzi et al., 2020). Furthermore, it is especially toxic to children, which can absorb ~40–50% of ingested Pb compared with only 3–10% in adults (Sevak et al., 2021). Intelligence deficits in children have been associated with blood Pb levels exceeding the concentrations of 75 mg/L and is linked with the developmental abnormalities (WHO and EFSA acceptable limit for Pb is 25 mg per kg of body weight) (EFSA Panel on Contaminants in the Food Chain, 2010). In addition, Pb is also a classified teratogen and mutagen and is comprehended to cause miscarriages, infertility, induce renal damage and genetic abnormalities in developing fetuses (Matta and Gjyli, 2016; Naik and Dubey, 2013). Its effects, however, are not just limited to its own sequestration site but go as far as the skeletal system, where it interferes with bone formation and acts like a long-lived toxicity stored here for 15–20 years (Vats et al., 2022; Sevak et al., 2021).

Furthermore, Pb pollution can also disrupt the ecological systems, thereby greatly changing the microbial community dynamics because soil microbial communities are Pb-sensible, which is extremely important for maintaining soil health and quality (Ma et al., 2020; Sevak et al., 2021; Fajardo et al., 2019; Li et al., 2020). Although Pb pollution reduces the abundance of soil microbes, it shifts these communities’ functional and phylogenetic composition and impairs soil physicochemical properties, thus degrading soil productivity and the resilience of ecosystems. Bacteria previously isolated from a Pb-polluted sight (e.g., *Acinetobacter junii* Pb1, *Bacillus subtilis* X3, *Delftia tsuruhatensis*, *Halomonas* sp., *Pseudomonas aeruginosa* N6P6) have developed Pb-resistant mechanisms, and were indicated to offer potential biotechnological applications for Pb bioremediation (Sevak et al., 2021). On the other hand, Pb pollution has its role in co-selecting AMR (Vats et al., 2022; Gupta et al., 2022) and studies have shown that lead can influence the prevalence of ARGs by creating selective pressures in microbial communities such as increased nasal colonization by the methicillin-resistant *Staphylococcus aureus* (MRSA) (Vats et al., 2022; Eggers et al., 2018).

#### 4.5.2 Mercury (Hg)

Mercury (Hg) is one of the most toxic naturally occurring elements posing a risk to ecosystems and human health (Ward et al., 2010; EFSA Panel on Contaminants in the Food Chain, 2012). Mercury is a widespread environmental contaminant released into the environment by natural geological processes (e.g., volcanic eruption or soil erosion) and human practices (e.g., mining, fuel combustion or industrial discharge) (Bazzi et al., 2020). Moreover, the region’s prevalence of mercury is further compounded by localized or regional crises, such as war, in the Middle East, which have caused conflict-induced pollution of especially high levels of contamination (Gworek et al., 2017).

Chemically, it comes in two main forms: inorganic (Hg 2^+^) and organic (methylmercury, CH_3_-Hg^+^), both being very toxic and can cause severe deleterious effects in humans even at low trace concentrations (e.g., neurological damage, kidney failure, and developmental defects) (Bazzi et al., 2020). Although Hg has no biological utility, it exerts ongoing selective pressure on microbe communities, forcing highly sophisticated resistance mechanisms to evolve (Vats et al., 2022; Sodhi et al., 2023). Their adaptations focus on the *mer* operon, a plasmid-encoded genetic cluster that enables bacteria to detoxify and resist Hg toxicity (Bazzi et al., 2020). The operon, responsive to the MerR protein, comprises a plethora of proteins, including the enzyme mercury (II) reductase (MerA), which converts the toxic form Hg^2+^ to its less toxic elemental form (Hg^0^). Of note, the *mer* operon has been found in numerous bacterial genera, including *Pseudomonas*, *Enterobacteriaceae*, and *Shewanella*, and is frequently borne on transposable elements to promote HGT (Vats et al., 2022; Sodhi et al., 2023). In avian *E. coli* strains, researchers have observed the co-location of Hg resistance genes with ARGs on MGEs, such as the Tn21 transposon, which provides the genetic linkage required for the co-transfer of resistance traits, with Hg exposure indirectly selecting for ARB (Vats et al., 2022; Biswas et al., 2021; Sodhi et al., 2023). For example, the co-selection of streptomycin resistance based on acquisition during exposure to Hg compounds has been previously detected. The transfer of Hg, *β*-lactams, and quinolone resistance was studied in ESBL-containing isolates from the Yamuna River, India, where plasmid recipients showed a co-resistance mechanism (Gillieatt and Coleman, 2024). Conjugation from Hg-resistant and As-resistant isolates into *E. coli* led to the acquisition of metal and antibiotic resistance, with transconjugants receiving ESBL, *mer*B, *mer*P, and *mer*T, including *qnr*S (Gillieatt and Coleman, 2024).

Other studies demonstrate that Hg as well as other HMs contamination can aggravate AMR in complex microbiomes, such as wastewater and soils, by triggering increased abundance of integrons and ARGs in environmental microbiomes, thereby accelerating the HGT of AMR traits (Frossard et al., 2018; Yazdankhah et al., 2018; Balcha et al., 2023). Additionally, it is possible that, with increasing global temperatures, methylmercury bioaccumulation in aquatic ecosystems may grow, increasing selective pressure via microbial communities (Jeong et al., 2024). With rising temperatures, the dissolved oxygen content in water declines, thus causing hypoxia, which favors the activity of anaerobic bacteria that cause Hg methylation and, therefore, advances methylmercury (CH_3_-Hg^+^) production (Jeong et al., 2024).

#### 4.5.3 Arsenic (As)

The term “arsenic” often provokes fear in many individuals. This reaction has been derived from its historical use as a poison—both deliberate and accidental—against humans (Hughes et al., 2011). Generally, arsenic (As) is sourced from natural and human activities using pesticides and herbicides, including agriculture (Bazzi et al., 2020). This trace element, is naturally found in the Earth’s crust at about 5 μg/g, comprising ≈ 245 minerals, and is often associated with HMs like Pb, Cu, Zn, and Au in sulfidic ores (Shen et al., 2013). The most hazardous and biologically active inorganic As forms are arsenite (As^3+^) and arsenate (As^5+^), whereas As^3+^ is more soluble and toxic due to its ability to bond with protein thiol groups (Chillé et al., 2022). While less toxic, organic As compounds often result from industrial or agricultural activities and can exhibit a wider range of toxicity effects.

As have left a significant pathway in history with the main role attributed as a weapon in warfare. Its extensive impact on the environment and public health was evident through the chemical agent Lewisite (C_2_H₂AsCl_3_), Adamsite (C_12_H_9_AsClN) and the herbicide Agent Blue, sizably used during the Vietnam War (Bazzi et al., 2020). Drawing from these historical insight, As contamination remains a critical problem with global consequences, particularly in South Asia (e.g., Afghanistan, Bangladesh, India, Nepal, Pakistan, and Sri Lanka), where millions of people are exposed to As concentrations in groundwater that exceed safe limits, leading to serious health issues such as multiple organ dysfunction, cancers, cardiovascular diseases, and neurodevelopmental disorders (Palit et al., 2019; Natasha et al., 2020; Mohammed Abdul et al., 2015). Furthermore, the utilization of these chemical biochemical weapons in the Syrian Civil War previously established by United Nations endued in expanded bacterial resistance to As mainly caused by oxidation, decreased methylation, activation of efflux pumps, and sequestration of cysteine -rich peptides (Bazzi et al., 2020; Shen et al., 2013). While, in agricultural setup, As-based compounds could worsen the AMR crisis through poultry feed additives like roxarsone (4-Hydroxy-3-nitrophenyl arsonic acid), which overuse of this led to increase of As content in manure, soil, and water, and was reported to facilitate As-resistance and ARB (Oremland and Stolz, 2005; Nachman et al., 2013).

As exposure influences AMR by acting as a selective pressure on microbial populations, leading to the simultaneous co-selection of resistance traits. The *ars* operon governs As resistance through efflux systems that expel arsenite (As^3+^) from bacterial cells (*E. coli* and *Shewanella*), mitigating its toxic effects (Silver and Phung, 2005). Recent studies indicate that ARB quickly co-selected after exposure to 0.2–1 mg/L As^3+^ for only 6 h, alongside augmented ARGs and MGEs. Most co-selected ARB persisted for at least 4 days without As^3+^ and antibiotics, suggesting that source water pollution may aid in preserving and spreading AMR determinants in the DWTP (Zhang et al., 2020). Analysis of bacterial community structure revealed a strong link between community shifts and ARB promotion, with the respective enrichment of opportunistic bacteria (e.g., *Escherichia-Shigella*, *Empedobacter* sp., and *Elizabethkingia*) (Zhang et al., 2020). Similarly, it was also shown that HMRGs and MGEs increased ARGs in As-contaminated soils under sulfamethoxazole stress (Li et al., 2023). In their study, copiotrophic *Actinobacteriota* abundance decreased, while oligotrophic *Gemmatimonadota* abundance expanded, indicating a shift in community strategy. *Gemmatimonadota* positively correlated with ARGs, HMRGs, and MGEs, suggesting it hosts resistance genes in As and SMX stress environments. The study concluded that MGEs play a key role in ARG proliferation via HGT and a co-selection mechanism, with bacterial communities indirectly influencing MGEs through environmental changes (Li et al., 2023).

#### 4.5.4 Chromium (Cr)

Chromium (Cr) is the 7th most abundant HM in the Earth’s crust and exists primarily in two stable oxidation states: trivalent chromium (Cr^3+^) and hexavalent chromium (Cr^6+^) (Bazzi et al., 2020). It was estimated that ~1.29 × 10^5^ t of Cr is discharged yearly into the environment and is mainly accumulated in the soil, thus causing severe Cr pollution (Ao et al., 2022). While Cr^3^+ occurs naturally in soil, water, and biological systems, Cr^6+^ is predominantly anthropogenic, originating from industrial activities (i.e., mining, leather tanning, electroplating, textile dyeing, and steel manufacturing) (Bazzi et al., 2020). The Cr^6+^ is highly soluble, very mobile, and approximately a thousand times more toxic than Cr^3+^ due to its strong oxidative capacity and metabolic reprogramming (Ahemad, 2014; Deng et al., 2019). Ingestion of Cr-contaminated water or food can lead to severe gastrointestinal symptoms, including nausea, vomiting, ulcers, and hemorrhage (Reif and Murray, 2024; Yan et al., 2023). The Cr^6+^ is rapidly absorbed through the gastrointestinal tract, and its accumulation in the liver and kidneys causes nephrotoxicity and impaired renal function (Wu et al., 2020). Chronic exposure has been linked to kidney failure due to its ability to disrupt tubular function and induction of oxidative stress (Verma et al., 2021).

The toxic nature of Cr^6+^ creates significant environmental and health risks, specifically in industrially contaminated sites. For example, elevated Cr-associated compounds have been reported in textile wastewater, landfill leachates, road runoffs and other industrially contaminated sites, contributing to environmental pollution and microbial stress (Tamburini et al., 2023; Ertani et al., 2017; Quiroz et al., 2023). The ongoing contamination with Cr and other HMs is particularly intense in regions with inadequate waste management systems, such as South Asia and the Middle East, where industrial effluents often remain untreated (Liu et al., 2024; Jiao et al., 2021). Similarly, other HMs, although not metabolically required, Cr exerts selective pressure on microbial populations, contributing to the evolution of sophisticated resistance mechanisms. These adaptations, often plasmid-mediated, can overlap with antibiotic resistance mechanisms, enhancing ARGs’ potential for cross-resistance and co-selection. Efflux pumps, particularly CHR family transporters, actively export Cr^6+^ ions out of bacterial cells, reducing intracellular toxicity. For example, the *chr*A gene in *P. aeruginosa* and *Shewanella* encodes a chromate efflux pump, providing resistance to Cr^6+^ while conferring MDR through cross-regulation (Joutey et al., 2015; Alvarez et al., 1999). Furthermore, *Bacillus cereus* co-contamination with Cr^6+^ and antibiotics was demonstrated to induce overexpression of eight assembled genes of the HAE-1 family of efflux pumps, favoring the emergence and spread of ARB (Wu et al., 2024).

#### 4.5.5 Cadmium (Cd)

Another toxic HM—Cadmium (Cd), with no essential biological role, has become a common pollutant all over the globe due to various anthropogenic activities. Historically, Cd contamination has escalated principally through mining, smelting, battery production, and phosphate fertilizers, which pose a consequential risk to ecosystems and human health because it is non-biodegradable, persistent, and has a bioaccumulative capacity (Chirinos-Peinado et al., 2022; Mortensen et al., 2018; Park et al., 2021; Sharma et al., 2024). Its toxicity has been associated with numerous adverse effects, including pulmonary/renal damage or even multiple organ damage, flu-like symptoms, and the infamous “itai-itai” disease, a painful condition linked to chronic Cd exposure in humans (Pu et al., 2021; Fatima et al., 2019). These deleterious effects are also extended to plant and animals, while bacterial and fungal agents have evolved and bear a significant resilience to increased Cd concentrations via the intricate defending mechanisms (Sharma et al., 2024).

Released into the environment, Cd significantly affects ecological compartments and strongly interacts with ARG dynamics (Shu et al., 2024). While in soils, Cd contamination was reported to alter microbial community composition, favoring *Proteobacteria*, *Luteimonas*, and *Bacteroidetes* while disrupting the environmental balance (Fu et al., 2023; Shu et al., 2024). The observed shift correlated with increased ARG abundance, particularly genes such as *tet*G, *tet*W, *sul*-1, and *sul*-2, further reproduced by MGEs like *intI*-1 and *intI*-2 (Anedda et al., 2023). For example, Cd exposure promoted the transfer of potential human bacterial pathogens such as *Clostridium* and *Burkholderia* in lettuce tissue and increased the abundance of ARGs (e.g., *erm*Q, *erm*X, *tet*G, *tet*C, *tet*W, *tet*X, *sul*-1, and *sul*-2) and the integrase gene *intI*-1 in oxytetracycline-polluted and non-polluted soil (Fu et al., 2023; Guo et al., 2021). Other recent experiments with bacterial cultures further confirmed that Cd exposure induced the activation of transmembrane efflux pump systems, conferring additional resistance to Zn and carbapenem antibiotics, emphasizing the cross-resistance mechanism (Goswami et al., 2023). In rhizosphere soils, the co-contamination of Cd with other HMs, such as Cu, worsens the co-selection of ARGs and HMRGs, facilitating the spread of MDR (Shu et al., 2024; Pan et al., 2023). Whereas, Cd in water environments could interact with other pollutants, including Fe₂O₃, creating synergistic effects that amplify its toxicity (Pu et al., 2021). It has been demonstrated that co-exposure to Cd^2+^ and nano Fe_2_O_3_ significantly enhanced the conjugative transfer of ARGs via the RP4 plasmid from *Pseudomonas putida* KT2442 to the microbial community of water microcosms (Pu et al., 2021). This co-exposure was reported to increase cell membrane permeability, elevate antioxidant enzyme activity, and upregulate expression of conjugative transfer genes, mechanisms that raise concerns about Cd′s role in the HGT of resistance traits. The study revealed that the majority of transconjugants were identified in human pathogens or opportunistic pathogens, including *Aeromonas veronii*, *Acinetobacter tandoii*, *Escherichia hermannii*, *Shigella boydii*, *Kluyvera cryocrescens*, *Vogesella perlucida*, *Klebsiella pneumoniae*, *Shewanella xiamenensis*, *Ralstonia mannitolilytica*, and *Serratia marcescens* underlining the potential risks associated with HMs, nanoparticles, and ARGs dissemination in water ecosystems, illustrating how Cd and other pollutants heighten AMR (Pu et al., 2021; Stepanauskas et al., 2006). Another example of how Cd exposure might indirectly increase methicillin resistance was the presence of Cd resistance (*czr*) and methicillin resistance (*mec*A) genes on the staphylococcal cassette chromosome mec (SCCmec) in *S. aureus* (Vats et al., 2022; Cavaco et al., 2010). Interestingly, Cd can change microbial resistance mechanisms in complex and antagonistic ways (Li et al., 2016). While higher Cd concentrations can inhibit the conjugative transfer of ARGs by inducing protective extracellular polymeric substances (EPS) in bacteria, environmentally relevant concentrations often drive synergistic interactions with antibiotics (Li et al., 2016). For example, the co-existence of Cd and enrofloxacin complex (Cd-EFX) has been reported to enrich Cd uptake in earthworms, illustrating how complexation reactions can amplify the combined toxicity of these pollutants (Li et al., 2016). Thus, Cd is a substantial pollutant due to its environmental persistence, biological toxicity, and involvement in AMR, as evidenced by pathogens such as MRSA and *Pseudomonas* spp.

#### 4.5.6 Nickel (Ni)

Nickel (Ni) is a naturally occurring HM in soil and while it is essential in trace amounts in particular biological functions, it poses influential environmental and health challenges when present in elevated concentrations (Das et al., 2018; Bartzas et al., 2021). Expanded industrial activities, particularly mining and smelting, have increased Ni pollution in different environments (Bartzas et al., 2021). Ni and its compounds are recognized as immunotoxic and carcinogenic agents (Guo et al., 2020), contributing to health issues, ranging from respiratory ailments, skin conditions to inflammatory conditions (Das et al., 2018; Bartzas et al., 2021; Guo et al., 2020; Genchi et al., 2020). A recent study examining the negative environmental impact of Ni production in Slovakia accentuated the socio-economic and political challenges in addressing the ecological burdens caused by Ni pollution (Levická and Orliková, 2024).

Ni could serve as a cofactor for several microbial enzymes essential for the virulence of certain pathogens. Notably, Ni-dependent enzymes such as urease and hydrogenase contribute to the pathogenicity of *Helicobacter pylori*, as well as *S. enterica Typhimurium* and *Staphylococcus* spp. which relies on these enzymes (Ni-acireductone dioxygenase, Ni-SOD, and Ni-glyoxalase I) for colonization and survival in the acidic environment of the human stomach (Maier and Benoit, 2019). Efficient Ni transport and homeostasis mechanisms are essential for these pathogens to balance the availability of Ni for enzyme function while mitigating its potential toxicity (Maier and Benoit, 2019). Furthermore, the presence of Ni ions can influence bacterial susceptibility to antibiotics, as was shown recently (Pavlić et al., 2022). Ni can interact with antibiotics such as ciprofloxacin and ampicillin, affecting their efficacy against bacteria like *S. aureus*, *E. faecalis*, and *E. coli*. Others have shown recently, the significant associations between Ni and bacterial resistance to tetracycline, sulphonamide, beta-lactams, aminoglycoside, macrolide, and vancomycin in the food producing environments emphasizing the need for new comprehensive environmental holistic management strategies (Anedda et al., 2023). The overall co-selection of HMs can lead to the proliferation MDR, complicating treatment strategies and posing significant public health challenges (Gillieatt and Coleman, 2024).

## 5 Conclusion

Taken together, HMs, particularly Pb, Hg, As, Cr, Cd, and Ni, are ubiquitous co-selective agents for the ARGs and MRGs in varied soil and water environments. Consequently, microbial community structures, HGT, and integron-mediated gene transfer are all amplified in the spread of AMR. Efforts to remediate HM concentrations in the surrounding matrix could indirectly reduce the toxic potential of HMs and the proliferation of ARGs. AMR proliferation in contact with microplastic pollution becomes more reasonable. Microplastics are novel substrates that carry and transport MRGS and ARB, allowing the localized ‘hot spots’ of gene exchange. The convergence of HMs pollution, MPs accumulation and pathogenic AMR bacteria constitutes concomitant vectors for spreading resistance, which offers extensive challenges to environmental management and public health. Determining metal pollutants should be investigated alongside steps to prevent MP contamination to ultimately inform integrated broad stroke mitigative efforts to protect the ecosystem and reduce AMR threat.
